# Biallelic germline MBD4 mutations predispose to colorectal polyposis, hypermutated AML, and schwannomas

**DOI:** 10.1007/s10689-026-00566-z

**Published:** 2026-05-08

**Authors:** Julia Cooper, Brittany L. Stewart, Lauren Bokovitz, Hetty Carraway, Sudipto Mukherjee, David Liska, Sumithira Vasu, Harry Lesmana, James S. Blachly

**Affiliations:** 1 Division of Human Genetics, Department of Internal Medicine, The Ohio State University, Columbus, OH USA; 2 Department of Medical Genetics and Genomics, Cleveland Clinic, Cleveland, OH USA; 3 Department of Hematology and Medical Oncology, Taussig Cancer Institute, Cleveland Clinic, Cleveland, OH USA; 4 Department of Colorectal Surgery, Digestive Disease Institute, Cleveland Clinic, Cleveland, OH USA; 5 Present Address: Division of Hematology, Department of Internal Medicine, The Ohio State University, Columbus, OH USA; 6 Present Address: Division of Pediatric Hematology, Oncology and BMT, Department of Pediatrics, Cleveland Clinic, Cleveland, OH USA; 7 Department of Biomedical Informatics, The Ohio State University, Columbus, OH USA

**Keywords:** MBD4, MANS, Polyposis, AML, Hereditary

## Abstract

Biallelic pathogenic variants in the *MBD4* gene have recently been associated with an autosomal recessive cancer predisposition syndrome deemed “*MBD4*-associated neoplasia syndrome (MANS)”. Early reports of individuals with MANS highlight the propensity to develop young onset colorectal polyposis and hyper-mutated acute myeloid leukemia (AML). In the following case series, we present four patients with MANS from three families with polyps and AML, as well as additional features previously less described, such as schwannoma and thyroid cancer. This series increases the total number of reported cases by 27%, with the intention of achieving a more robust understanding of the full phenotypic spectrum of MANS.

*MBD4* (Methyl-CpG Binding Domain 4)*,* a gene encoding a DNA glycosylase functioning in base excision repair, has recently been described in association with autosomal recessive predisposition to early-onset acute myeloid leukemia (AML) and colorectal polyposis [1–7]. Prior work demonstrates biallelic loss of *MBD4* facilitates cytosine-phosphate-guanine (CpG) to thymine-phosphate-guanine (TpG) mutations accumulate in the genome, predisposing to AML with a unique hyper-mutated genomic signature typically including biallelic hits to *DNMT3A* [1, 6]. Recent publications have deemed this “*MBD4*-associated neoplasia syndrome (MANS)”. A more complete understanding of the full phenotypic spectrum and tumorigenic mechanisms of MANS is critical for early recognition, diagnosis, management, and treatment. Here, we present four patients with MANS from three families seen at two referral centers in Ohio, United States of America.

*Case 1.* A 28-year-old female presented to a local emergency department with 81% circulating blasts. One month earlier, she underwent trans-anal excision of a recurrent 3 cm tubulovillous adenoma in the distal rectum, initially present at age 21. Her recovery was unusually slow and painful, and subsequent immunophenotypic examination of her blood yielded a diagnosis of AML. Genetic testing of leukemic blasts revealed a hypermutated profile dominated by numerous CpG to TpG transitions (Table 1). Fluorescent in-situ hybridization (FISH) studies for abnormalities common in AML were negative and her metaphase karyotype demonstrated 46,XX[20]. She started standard intensive cytarabine and daunorubicin induction chemotherapy (“7 + 3”); her course was complicated by neutropenic fever, acute pain, cellulitis, rash, and bacteremia, resulting in multiple re-admissions. Two months after beginning induction chemotherapy, a bone marrow biopsy showed 1% myeloblasts and no evidence of disease, with improving peripheral blood counts.

**Table 1:** Available somatic mutation analyses

Somatic testing	Driver gene	Pathogenic variant	VAF	Other findings
*Case 1*				
Acute myeloid leukemia (bone marrow)	*BCOR*	c.3649C > T p.R1217*	38.7%	Includes CpG > TpG SNV (classified as VUS) in *RUNX1* c.1076C > T, p.(Pro359L) at 44.5% VAF on a 51 gene panel
	*BCORL1*	c.1825C > T p.R609*	46.5%	
	*DNMT3A*	c.2645G > A p.R882H	44.5%	
	*IDH2*	c.419G > A p.R140Q	43.2%	
	*WT1*	c.1372C > T p.R458*	4.2%	
	*WT1*	c.1288C > T p.R430*	7.6%	
*Case 2B*				
Vestibular schwannoma (right)	*NF2*	c.1396C > T p.Arg466*	13.4%	High TMB at 16.8 m/Mb (94th percentile) Includes CpG > TpG SNV (classified as VUS) in 16 additional reported genes (out of 29 total additional gene variants on a 648 gene panel)
	*NF2*	c.784C > T p.Arg262*	12.2%	
Acute myeloid leukemia (bone marrow)	*DNMT3A*	c.2644C > T p.Arg882Cys	20.5%	SNP array showed copy loss of heterozygosity in 1p and 1q Includes CpG > TpG tier 2 mutations in *BRINP3, WT1, KMT2A, BCOR*, and *SMC1A* on a 68 gene panel
	*TET2*	c.4546C > T p.Arg1516*	20.8%	
	*CSF3R*	c.2308C > T p.Gln770*	34.2%	
	*ETV6*	c.641C > T p.Pro214Leu	20.9%	
	*RUNX1*	c.602G > A p.Arg201Gln	18.7%	
	*STAG2*	c.775C > T p.Arg259*	22.2%	
	*CBL*	c.1259G > A p.Arg420Gln	40.2%	
Duodenal Tubular Adenoma	*APC*	c.847C > T p.Arg283*	18.4%	High TMB at 24.2 m/Mb (96th percentile) Includes CpG > TpG SNV (classified as VUS) in 25 additional genes (out of 26 total additional gene variants on a 648 gene panel)
	*APC*	c.4348C > T p.Arg1450*	18.3%	
	*RAF1*	c.770C > T p.Ser257Leu	21.0%	
	*KRAS*	c.40G > A p.Val14lle	19.4%	
	*CTCF*	c.1130G > A p.Arg337His	16.3%	
	*INPP4B*	c.889C > T p.Arg297*	12.4%	
	*MAP2K1*	c.199G > A p.Asp69N	6.2%	
	*SMARCA4*	c.3575G > A p.Arg1192His	3.2%	
Thyroid Cancer	*BCORL1*	c.3142C > T p.Arg1048*	40.2%	High TMB at 27.4 m/Mb (97th percentile) Includes CpG > TpG SNV (classified as VUS) in 12 additional genes (out of 13 total additional gene variants on a 648 gene panel)
	*CIC*	c.3331C > T p.Arg1111Trp	29.2%	
	*HRAS*	c.182A > G p.Gln61Arg	27.7%	
	*PTPN11*	c.3G > A p.Met1?	26.1%	

During 7 + 3, genetics was consulted given her young age at diagnosis and history of an early onset advanced colon polyp. Family medical history (Fig. 1A) was noncontributory. There was reportedly one distant maternal relative with older onset “leukemia.” The patient’s youngest brother was identified as a full HLA match and preferred hematopoietic stem cell (HSCT) donor. Germline testing, performed on skin fibroblasts, interrogated 61 genes associated with acute leukemia and bone marrow failure syndromes. Testing revealed two *MBD4* variants, c.939dup, p.(Glu314Argfs*13) and c.614_615del, p.(Ser205*). These biallelic truncating variants in *MBD4* provide a unifying explanation for her early-onset colorectal neoplasia, hypermutated AML and lack of family history. Cascade testing was performed, prioritizing the fully HLA-matched brother; he tested negative for both variants. Her other two brothers were each heterozygotes bearing c.614_615del, p.(Ser205*) and negative for c.939dup, p.(Glu314Argfs*13).

**Fig. 1:**
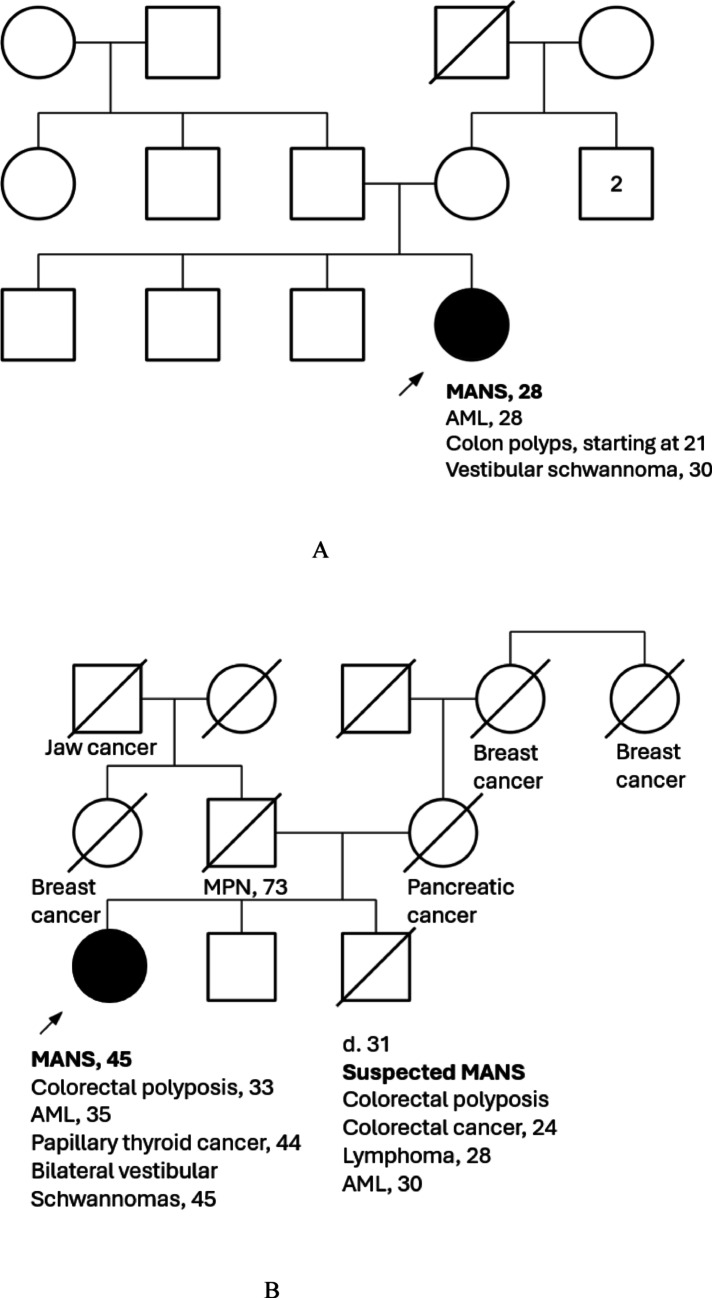

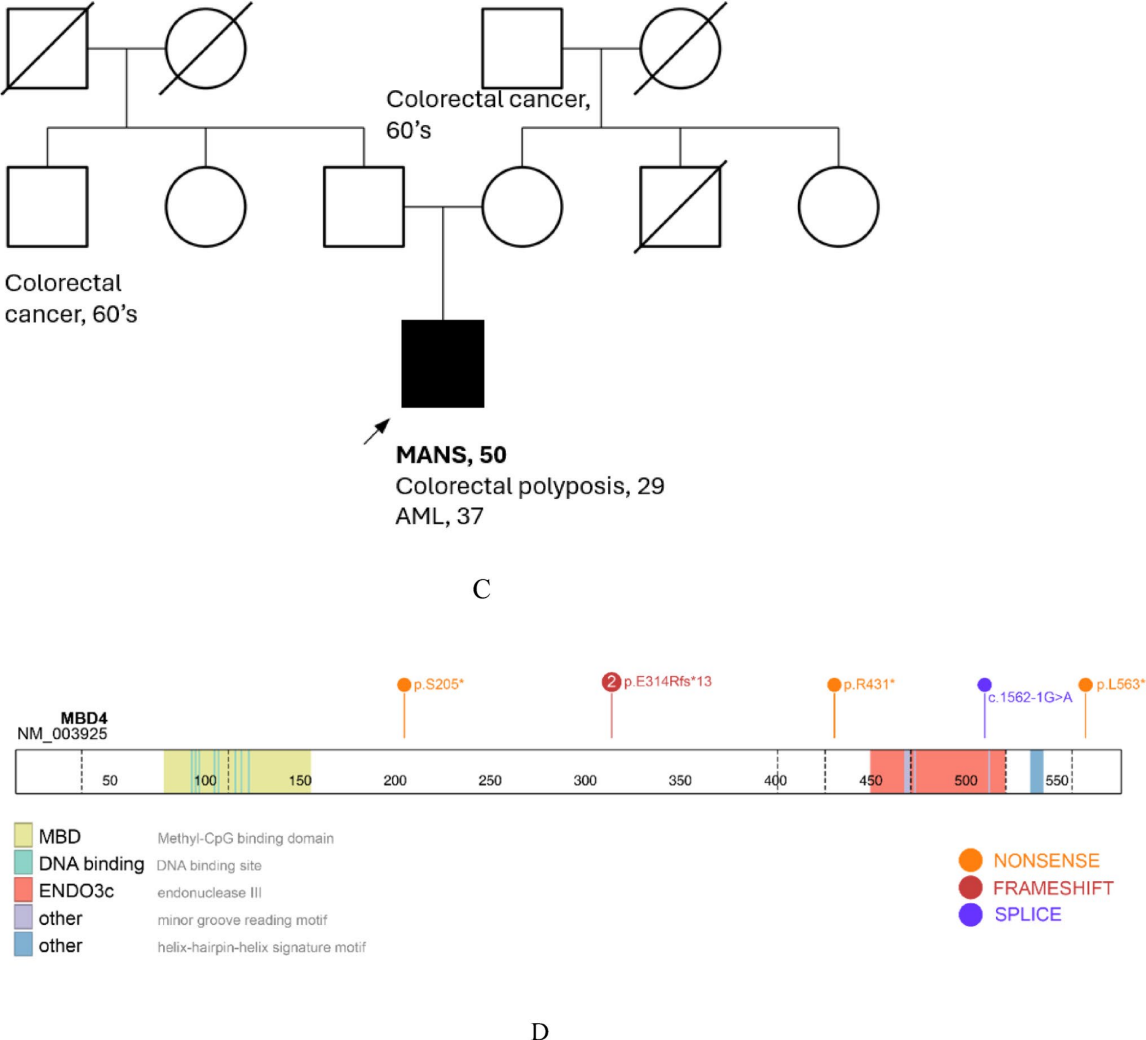
Pedigrees from cases 1 (Fig. 1A), 2A and 2B (Fig. 1B), and 3 (Fig. 1C); and a lollipop plot depicting 5 distinct mutations in *MBD4* across 3 compound heterozygous patients (Fig. 1D). **A** Pedigree from case 1. **B** Pedigree from case 2A and 2B. **C** Pedigree from case 3. **D** Lollipop Plot. MANS: *MBD4*-associated neoplasia syndrome; AML: acute myeloid leukemia; MPN: myeloproliferative neoplasm

After successful transplant, the patient had a brain MRI for headaches, revealing a previously unidentified vestibular schwannoma. She has since undergone two colonoscopies, revealing two and three tubular adenomas, respectively.

*Case 2A and 2B.* Two siblings (brother and sister) from a non-consanguineous family with strong family history of early onset and diverse malignancies (Fig. 1B) were diagnosed with early-onset AML and colorectal polyposis in their 30 s. Prior to the diagnosis of AML, the brother (2A) was diagnosed with colorectal cancer and lymphoma at 24 and 28 years old, respectively, and treated with multimodal therapy including surgery, chemotherapy, and radiation. He was diagnosed with AML at 30 years and underwent allogenic-HSCT using his sister as donor but subsequently passed away following relapse of his AML. Neither somatic nor germline genetic testing had been completed for him. The sister (2B) had a clinical diagnosis of familial adenomatous polyposis (FAP) at 33 years and underwent a total abdominal colectomy with ileorectal anastomosis due to numerous (> 70) hyperplastic and adenomatous colorectal polyps. Now age 49, she continues close monitoring for adenomatous polyposis with upper endoscopy and sigmoidoscopy, which has revealed multiple adenomas of the rectum and small bowel including innumerable adenomas in the ileum and greater than twenty duodenal adenomas.

At age 35, she was diagnosed with AML. She received two cycles of 7 + 3 and achieved remission prior to allogenic-HSCT using matched unrelated donor. Her post-transplant course was complicated by chronic graft versus host disease treated with tacrolimus. She remains in remission post-HSCT. She then underwent total thyroidectomy at age 44 revealing papillary thyroid carcinoma. Shortly after, she was evaluated with brain MRI due to sensorineural hearing loss, which identified bilateral vestibular schwannomas leading to a clinical diagnosis of *NF2*-related schwannomatosis. Her right schwannoma was resected and the smaller, left schwannoma is actively monitored. Due to concern for germline cancer predisposition syndrome, whole exome sequencing was performed and identified two *MBD4* variants in trans: c.1291C > T, p.(Arg431*) and c.1688 T > A, p.(Leu563*). Importantly, no germline variants were detected in *APC* or *NF2*, ruling out molecular diagnosis of FAP and *NF2*-related schwannomatosis, respectively. To further assess for MANS, clinical somatic tumor profiling was either reviewed or if not yet completed, performed for any of the patient’s neoplasms for which tissue was available. Multiple acquired CpG > TpG single nucleotide variants (SNVs) were identified on somatic testing of the patients AML, thyroid cancer, right vestibular schwannoma, and a 25 mm duodenal tubular adenoma that was excised when the patient was age 45 (Table 1). This included pathogenic variants in driver genes consist with each neoplasm type including a *DNMT3A* mutation in the AML, biallelic *NF2* mutations in the schwannoma, and biallelic *APC* mutations in the duodenal adenoma.

*Case 3.* A 50-year-old male with history of adenomatous polyposis diagnosed at age 29 years and AML diagnosed at age 37 years, currently in remission. When he saw a genetic counselor in 2021, a multi-gene hereditary cancer and polyposis panel was ordered. Results from this initial testing were negative. He then underwent expanded panel testing, which included newer hereditary leukemia genes, with specific concern for *MBD4* given his personal history and lack of an obvious autosomal dominant predisposition syndrome in his pedigree (Fig. 1C). Expanded panel testing identified biallelic variants in *MBD4*: c.939dup, p.(Glu314Argfs*13) and c.1562—1G > A.

We report four patients from three families with MANS, increasing the total number of reported cases by to 19 (a 27% increase overall). Furthermore, we contribute a previously unreported variant, c.1562—1G > A. Each of our patients had AML diagnosed between the ages of 28 and 39 years. Two of the four had hypermutated disease; the remaining two were diagnosed before somatic testing was routine and therefore their mutational signature cannot be known. The hypermutated phenotype typically manifests as CpG > TpG transitions (note that when G > A is reported in the coding strand this still represents CpG > TpG on the opposite strand). This young-onset AML is consistent with previous reports of MANS-related AML. Of the 15 individuals with MANS associated AML previously reported in the scientific literature, 11 of them were diagnosed in their 20 s or 30 s [1–4, 6, 7]. Each of our four patients had pathologically confirmed adenomatous polyposis beginning in their 20 s, including one patient who had a recurrent tubulovillous adenoma at age 20. Adenomatous polyposis seems to be as highly penetrant as AML in people MANS [1–4, 6, 7]. However, the cumulative number of colon adenomatous polyps widely ranges among reports, regardless of an individual’s age. Blombery et al. previously reported one MANS patient with vestibular schwannoma; here, we report two patients from separate families who developed vestibular schwannomas further supporting schwannoma as a less penetrant feature of MANS [3]. Of note, Palles et al. described one patient with chest wall and cervical schwannoma, and Querido et al. reported one patient with schwannoma in an unspecified location [2, 7]. Genomic sequencing of the vestibular schwannoma in one of our patients revealed a high mutational burden (16.8 m/Mb, 94th percentile) and 16 total CpG > TpG transitions, which is a mutational signature of biallelic *MBD4* deficiency. This same patient had genomic sequencing of her papillary thyroid tumor which revealed a similarly robust CpG > TpG signature and high mutational burden (27.4 m/Mb, 97th percentile). The identification of biallelic somatic *NF2* mutations in the schwannoma and biallelic *APC* mutations in adenoma strongly supports *MBD4*-driven hypermutation as the mechanism underlying tumorigenesis across tissues. This is the first report of extensive sequencing of respective tissues in a person with MANS and supports that other types of cancer are potentially associated with biallelic germline mutations in *MBD4*. Of note, none of our three families had a known family history of uveal melanoma, which has previously been reported with heterozygous pathogenic mutations in *MBD4* and two individuals with MANS [2, 5, 8–11]. However, the exact risk in carriers is debated.

Historically, tumor and germline genetic testing laboratories have not routinely included *MBD4* on gene sequencing panels. Therefore, it is likely that many individuals, especially with early-onset AML, colon polyps and/or colorectal cancer, and lack of family history of cancer, remain unidentified. We endorse routine inclusion of *MBD4* on germline cancer predisposition panels. Individuals with previously unexplained phenotypes consistent with MANS need to be re-tested to rule out this newly described syndrome. Identifying these individuals will help to further characterize the spectrum of this syndrome, which likely includes solid tumors, as evidenced in Case 2. Furthermore, the National Comprehensive Cancer Network (NCCN) Clinical Practice Guidelines in Oncology now recommends consideration of *MBD4* testing in any person with > 10 lifetime adenomatous polyps [12]. For those with MANS, NCCN Guidelines recommend high quality colonoscopy starting at 18 to 20 years old and repeated every two to three years. NCCN also offer screening recommendations for AML and uveal melanoma, which includes complete blood count (CBC) at diagnosis and annual ophthalmology exam, respectively. Our data suggests that surveillance strategies for MANS may ultimately need to expand beyond these tissues to include nervous system and thyroid, although additional longitudinal data will be required to define risks and optimal screening intervals.

In summary, *MBD4* deficiency is an under-recognized DNA-repair disorder conferring high risk for early-onset myeloid and epithelial malignancies with hypermutated mutational signature when biallelically inactivated. The extreme mutational burden observed in MANS-associated tumors suggests potential sensitivity to immune checkpoint blockade as previously observed in MBD4-deficiency uveal melanoma [8]. Increased recognition of this syndrome will improve diagnosis, inform transplant decision-making, and enable rational surveillance strategies for affected individuals and their families.

Tumor mutational burden (referred to as “TMB” by the clinical laboratory that performed the somatic testing) is defined (by the performing laboratory) as the measure of the quantity of somatic SNVs and indels, of any pathogenicity, including benign, carried in a tumor calculated as the number of protein-altering mutations per million coding base pairs. The percentile for each TMB is calculated relative to all tumor samples sequenced at the performing laboratory. VUS: Variant of uncertain significance. m/Mb: mutation per megabase.

Please note that several CpG > TpG variants are reported as G > A mutations since the CpG > TpG deamination occurred on the strand opposite of the gene transcript.

## Data Availability

No datasets were generated or analysed during the current study.
